# Fundraiser engagement, third-party endorsement and crowdfunding performance: A configurational theory approach

**DOI:** 10.1371/journal.pone.0308717

**Published:** 2024-08-15

**Authors:** Qingxiang Li, Nianxin Wang

**Affiliations:** School of Economics and Management, Jiangsu University of Science and Technology, Zhenjiang, China; University of Gdansk Faculty of Economics: Uniwersytet Gdanski Wydzial Ekonomiczny, POLAND

## Abstract

Reward-based crowdfunding is a typical two-sided platform (fundraiser side and backer side) with high information asymmetry. While existing research indicates that signals from fundraisers and backers can impact crowdfunding performance, the interplay among these signals warrants further investigation. Drawing on signaling theory, this study adopts a configurational perspective and utilizes the fsQCA method and linear regression to investigate the combined effects of fundraiser engagement (update and fundraiser comment), third-party endorsement (backer comment and Facebook sharing), and project preparedness (video, image, and description) on crowdfunding performance. Drawing data from the reward-based crowdfunding platform Indiegogo, this research pointed out that these signals cannot generate better crowdfunding performance alone and examined substitution and complementary effects among different signals. Based on the linear regression and fsQCA results, configurations that lead to high crowdfunding performance are identified. We found that project preparedness must work with other signals to produce high crowdfunding performance. Besides, we summarized these configurations into two patterns that may lead to high crowdfunding performance: a fundraiser engagement-driven pattern and a third-party endorsement-driven pattern. This study contributes a configurational perspective and valuable insights into how signals can work together to mitigate information asymmetry in crowdfunding.

## Introduction

Crowdfunding platforms enable an initiative to collect contributions for their products or services through an open call on the internet by directly tapping the general public instead of choosing the traditional finance approach [1, 2]. As an emerging internet-based fundraising platform, it has grown rapidly. According to the report, the global crowdfunding market was valued at $10.2 billion in 2018 and would reach $28.77 billion by 2025 [3]. The entrepreneurial finance market includes four types of crowdfunding: reward-based, donation-based, lending-based, and equity-based. In this paper, we are particularly interested in understanding what leads to reward-based crowdfunding performance. In reward-based crowdfunding, project owners, also known as fundraisers, initiate the process by posting their creative ideas on platforms like Indiegogo. Subsequently, they appeal to individual investors, known as backers, to make small contributions to support their ventures.

As an emerging financing method, crowdfunding faces serious information asymmetry. This information asymmetry comes from two aspects: First, one party is not fully aware of the characteristics of another party or is uncertain about the quality of information. Second, information asymmetry is also significant when one party is concerned about another party’s behavior or behavioral intentions. Previous studies have used signaling theory to understand how to alleviate information asymmetry to help project financing. Existing research has indicated that signals from projects, fundraisers, and backers can affect the outcome of crowdfunding. For example, some studies pointed out that a fundraiser’s social capital [4, 5], engagement [6], and credibility [7] can affect crowdfunding performance. Some studies focused on the backer’s peer influence [6], social media activity [5, 8], and endorsement [9], which also plays an important role in fundraising. Besides, some studies uncovered that project characteristics as signals of preparedness may affect project performance, such as video [1, 10], images [5], and project description [11, 12].

Although plenty of research has generated many insights into determinants of crowdfunding performance, how these signals work together to influence fundraising performance remains underexplored. The reasons why those signals should be explored using a configurational perspective are threefold. First, single signals may complement each other [9]. For example, signals from fundraisers presenting more useful information may complement project-originating signals and synergistically affect crowdfunding performance [13]. Second, certain signals being present may compensate for the absence of others. For example, video has been proven as an important signal that may influence crowdfunding performance. If a video may give more detail about a project, those projects without video may need to rely on other signals. Third, potential backers perceived different trusts from different signals, which are interdependent [14].

Complex interactions among various signals influence crowdfunding performance. Using only regression models to analyze these signals is insufficient and may bring bias. There are complex interactions between backers and fundraisers, so the performance of crowdfunding projects is affected by the combined impact of these signals, not just any single one. Besides, research has called for exploring the combination of signals due to the interdependence between signals [15]. Therefore, we seek a configurational approach to assess the determinants of crowdfunding performance. As configurational theory focuses on combinations and synergies among multiple signals, it best applies to investigating this research’s complex nonlinear and asymmetric relationships [16]. Taken together, the limitations of existing studies give rise to our research question: How do signals configurationally affect the funding performance of projects in reward-based crowdfunding?

We adopted linear regression and fuzzy-set qualitative comparative analysis (fsQCA) to examine how fundraiser engagement, third-party endorsement, and project preparedness work together to affect crowdfunding performance. Specifically, we include updates (the number of updates posted by fundraisers), and fundraiser comments (the number of comments posted by fundraisers) as signals of fundraiser engagement. Third-party endorsement concerns the extent to which the project is endorsed by independent crowds, and we use positive comments (the number of positive comments posted by backers) and Facebook sharing (the number of Facebook shares of the project) as the signal of third-party endorsement. For project preparedness, we choose video (the number of videos of a project), image (the number of images of a project), and description (length of the project description) as the signals of project preparedness. We aimed to explore how these signals interplay with each other to affect crowdfunding performance.

To obtain the configurational pattern to achieve high project performance, we collect data from one of the largest reward-based crowdfunding platforms, Indiegogo. Using the linear regression and fsQCA method, we investigated the complementary effect or substitution effects between signals of fundraisers, projects, and backers to extend the existing literature. Our results identify two distinct patterns that may bring high performance, including (a) fundraiser engagement-driven pattern and (b) third-party endorsement-driven pattern. Besides, our results reveal that signals cannot lead to high performance solely, especially that project preparedness must work with other signals to achieve better outcomes.

Our work offers several important contributions to the crowdfunding literature. First, we adopt the configurational perspective and fsQCA approach to investigate the complex nonlinear relationship between different signals and crowdfunding performance. While prior research has offered valuable insights into determinants of crowdfunding performance [5, 13, 17, 18], the combination of signals has yet to be thoroughly explored. We extended this literature line by examining how the combination of multiple signals influences crowdfunding performance.

Second, we contribute to the studies about the effect of social media activities on crowdfunding performance [1, 19, 20]. In contrast to previous studies underscoring the significance of social media in crowdfunding performance [1, 19, 21], this research introduces a pattern for attaining high performance that does not heavily rely on social media. We identified that high fundraiser engagement and well-prepared projects can still achieve success in the absence of social media activities. This finding is important because it provides more possibilities for fundraisers with insufficient social capital.

Third, we extended the literature review on determinants of reward-based crowdfunding performance. After performing a thorough literature search and analysis, this paper details the basic characteristics of these studies, including publication year, quantity, and theoretical foundations. We further analyze studies that employ signaling theory to investigate the determinants of reward-based crowdfunding. The influencing signals are categorized from three perspectives: fundraisers, backers, and project attributes. This literature review aids researchers in understanding the findings of previous empirical studies and figuring out future directions.

## Literature review

### Information asymmetry in reward-based crowdfunding

Crowdfunding enables individuals to collect funds through an open call on the Internet, which directly taps into the general public instead of more traditional financing sources [1, 22]. Derived from microfinance [23] and crowdsourcing [24], crowdfunding has emerged as an innovative way of capital. The transition of innovative ideas from initial concepts to large-scale production inherently comes with risks and information asymmetry, making it difficult to assess the true value and potential of these ideas [2]. This discrepancy in knowledge, especially regarding the quality of a project or the reliability of its fundraiser, poses significant obstacles in the crowdfunding context [25]. Fundraisers often deeply understand their project’s merits, but potential backers might not have sufficient insight into the fundraiser’s trustworthiness or capability to fulfill promises regarding product delivery. The real value of the product remains hidden until it is in the hands of the backers, highlighting the issue of information asymmetry in crowdfunding ventures [1].

This information asymmetry creates considerable difficulties for fundraisers and backers. Backers, lacking the necessary information, may struggle to discern the potential and risks of various projects. Conversely, project fundraisers find it challenging to effectively communicate the value and prospects of their projects to attract investment. Therefore, developing and implementing strategies to minimize the information asymmetry between fundraisers and backers is crucial for the fruition of crowdfunding efforts.

To mitigate information asymmetry, existing research has generally explored reward-based crowdfunding. Signal theory is critical elements that may help reduce risks and uncertainties in online exchanges. Therefore, this paper will subsequently discuss three perspectives: studies on reward-based crowdfunding, signaling in reward-based crowdfunding.

### Studies on reward-based crowdfunding

To alleviate information asymmetry in crowdfunding, many scholars have attempted to explore the signals influencing crowdfunding performance from multiple perspectives to help the crowdfunding market develop rapidly and healthily. To grasp the current state and progress of existing research, this paper first provides a comprehensive literature review on the determinants influencing reward-based crowdfunding performance.

The Web of Science (WoS) Core Collection was used to obtain a representative dataset of determinants of crowdfunding literature. Various keyword combinations were used to obtain an exhaustive list of crowdfunding papers from WoS. Based on reading abstracts as well as a few widely cited articles, and after several tests, we designed the final search criteria using the words that related to crowdfunding (i.e., crowdfunding, crowd funding, crowd-fund, crowd fund, crowd funder, crowd-funder) combined with performance-related words (i.e., success, performance, outcome, succeed, successful, backer decision, investment decision). Searches were conducted on the titles, abstracts, and keywords.

The search resulted in 257 papers and we performed data cleansing to make it more relevant and focus on reward-based crowdfunding performance. First, we successively refined the data set based on WoS document type and discipline category. 13 papers were excluded; only journal and conference papers were retained. Second, we read the abstracts of all papers and manually removed 10 papers that contained “crowdfunding performance” in the text but did not investigate determinants of crowdfunding performance. After this further reduction, 234 papers remained. Third, since our research mainly focuses on the reward-based crowdfunding context, studies on other types of crowdfunding were excluded and 185 papers remain.

At this stage, the selected articles are carefully read. We manually extracted and coded into a database including the following elements: author, year, title, publication, sample (size and data source platform), research methods (empirical, experiment, case study, conceptual, etc.), the determinants of crowdfunding performance, the measurement of crowdfunding performance and the main findings. After data extraction and coding, we achieve a basic understanding of existing literature about determinants of crowdfunding performance.

**Fig 1:**
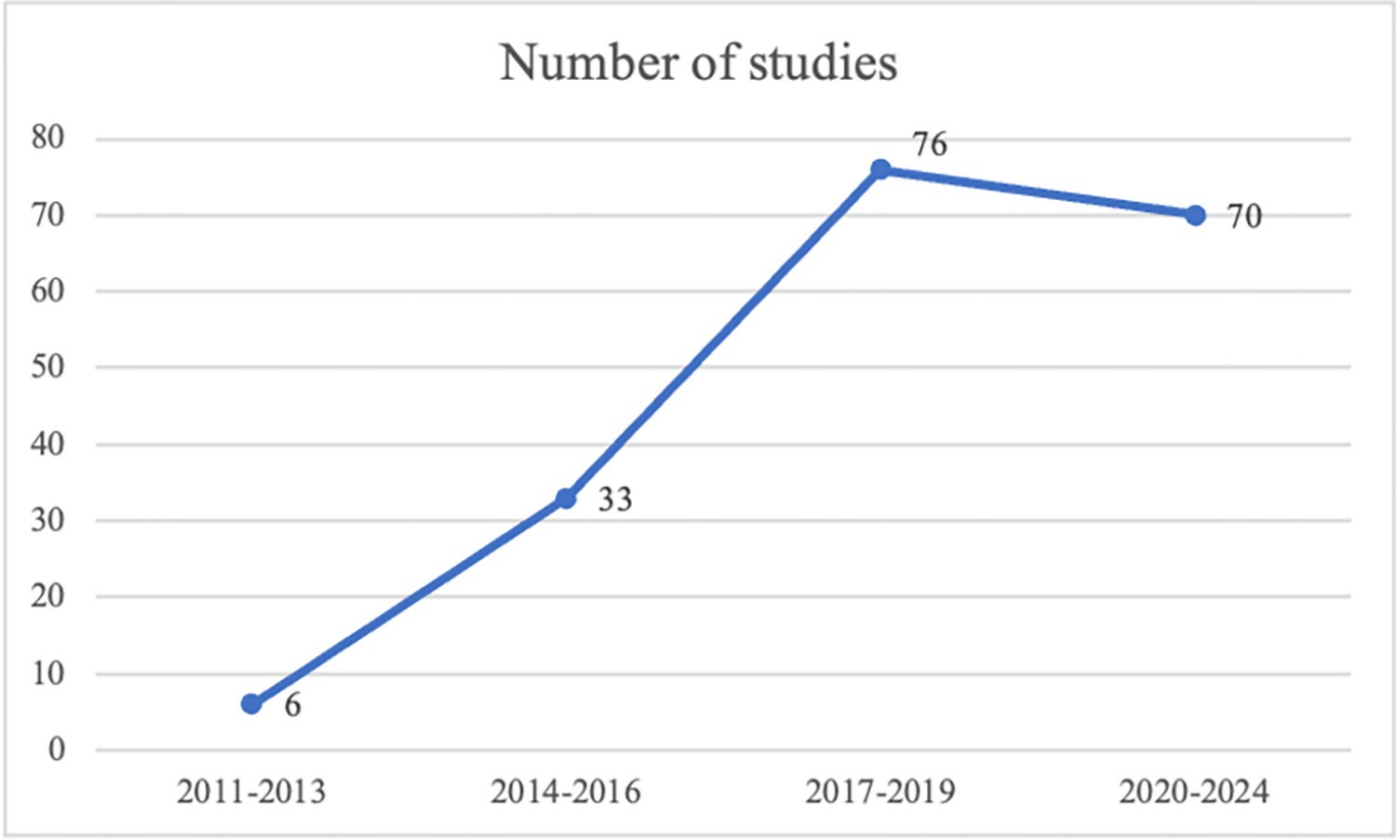
Temporal trends of studies.

First, we show the temporal trends of the selected papers. Fig 1 shows that research on determinants of crowdfunding performance has shown significant growth since 2014. Details are shown in Table 1.

**Table 1:** Temporal distribution of studies.

Year	#
2011	1
2012	1
2013	4
2014	6
2015	12
2016	15
2017	20
2018	31
2019	25
2020	31
2021	24
2022	7
2023	7
2024	1

Second, we found that most studies use secondary data from crowdfunding websites, with more studies using data from the Kickstarter platform (99 out of 185 papers), while exploration of the Indiegogo platform is relatively lacking. This also provides an opportunity for our research.

Third, existing literature has used a variety of theoretical perspectives to explore the signals influencing crowdfunding performance, including signaling theory, behavior theory, social capital, elaboration likelihood model, social identity theory, trust theory, language expectancy theory, and others. Table 2 lists all the theories used in selected papers (only including articles that explicitly state the specific theoretical basis in the full text).

**Table 2:** Theory basis of existing literature.

Theory	Studies	#
signaling theory	[4–10, 12, 13, 26–41]	25
behavioral theory	[42]	1
choice overload	[43]	1
stereotype content theory	[44]	1
observational learning and social influence	[45]	1
self-determination theory	[46]	1
event system theory	[47]	1
social network theory	[48, 49]	2
media richness theory	[50]	1
eWOM	[49, 51, 52]	3
social exchange theory	[53]	1
entrepreneurial learning	[54]	1
social capital	[55–63]	9
social proof	[64–66]	3
affective event theory	[11]	1
warm-glow theory	[8, 67]	2
cognitive load theory	[68]	1
framing theory	[69, 70]	2
consumption value theory	[71]	1
stimulus-organism-response (S-O-R) framework	[72]	1
brand management	[73]	1
advertising appeal	[74]	1
construal level theory	[75]	1
social role theory	[76]	1
regulatory focus theory	[77]	1
five factor model	[78]	1
elaboration likelihood model	[13, 31, 74, 79–83]	8
unimodel theory of persuasion	[84]	1
social norm	[85]	1
actor–resource–activity (ARA) model	[86]	1
social identity theory	[8, 38, 87, 88]	4
language expectancy theory	[20, 89]	1
critical theory	[90]	1
socioanalytic theory	[91]	1
social movement	[92]	1
phantom effect theory	[93]	1
trust theory	[10, 94]	1
motion effect theory and visual search theory	[72]	1
uncertainty reduction theory	[89]	1
goal-setting theory and narrative theory	[8, 95]	2
dual-process theory	[80, 96]	2
Theory of Planned Behavior (TPB)	[97]	1
unified theory of acceptance and use of technology (UTAUT)	[98]	1
social psychology theory	[99]	1
Theory of communication	[100]	1

Through the systematic literature review and analysis mentioned above, the relevant research on signals influencing the performance of reward-based crowdfunding has been summarized. It can be seen that numerous signals are affecting crowdfunding performance, including project attributes (such as duration [5, 101], goal [29, 79, 102], description length [12, 68], etc.), fundraiser characteristics (such as experience [50, 94], social network [5, 61], gender [103, 104], etc.), and backer behavior and characteristics (such as peer influence [6, 60], information hiding behavior [85], motivation [105–107], circadian [108], etc.). As shown in Table 2, signaling theory is a primary theoretical basis for exploring the signals influencing crowdfunding performance. Therefore, combined with our research purpose, this article will further focus on the research that uses signaling theory in the context of reward-based crowdfunding and summarize the specific determinants of reward-based crowdfunding performance.

### Signaling in reward-based crowdfunding

Signaling theory is fundamentally concerned with reducing information asymmetry between two parties [109]. In crowdfunding, information asymmetry may come from two aspects. First, one party is not fully aware of the characteristics of another party or is not sure about the information of quality. Second, information asymmetry also is important when one party is concerned about another party’s behavior or behavioral intentions [110]. Backers will evaluate projects based on signals from observable information to support their decision on whether to make contributions to projects. This decision-making process is consistent with signaling theory in terms of mitigating information asymmetry by available information to draw inferences on unobservable qualities or intentions [111].

Numerous empirical scholars have researched the impacts of signals on crowdfunding fundraising performance. After the above systematic literature review and analysis, we have compiled relevant studies (25 papers) on the signals affecting crowdfunding project success. S1 Table lists all 25 papers that use signaling theory to investigate determinants of crowdfunding performance and their main findings.

First, we summarize the research methods used in the 25 papers. Except for one study that constructed a theoretical framework [41], the remaining 24 studies are mostly empirical research based on secondary data. The most commonly used research methods are linear regression, logistic regression, and probit regression. Table 3 shows detailed information. Among these 24 papers, only 1 paper conducted the QCA method to investigate the interaction between signals on reward-based crowdfunding performance, which proves the necessity of this research.

**Table 3:** Research methods in the literature.

Method	Studies	#
regression (linear or OLS)	[12, 26–29, 31–35, 37–40]	14
logistic regression	[4, 5, 9, 10, 12, 13, 28, 29]	8
probit regression	[8, 9]	2
negative binomial panel	[6]	1
game theoretic model	[30]	1
functional data analysis	[26]	1
QCA	[7]	1
GMM	[36]	1

Note: some studies conducted more than one analysis method.

Second, from the 25 papers, we identified that signals influencing crowdfunding performance primarily come from three sources: projects-, fundraisers-, and backers-related signals. To better align with the research questions of each paper, the standard for inclusion is that only independent variables, mediator variables, and moderator variables in the research model are marked as valid signals and then be classified. If a factor is only used as a control variable, it is not recorded. It should be noted that the construct of the same factor may differ across studies. For example, *video* may be considered as a measure of a project’s preparedness [7] or as a measure of a project’s information disclosure [4]. Based on these 25 papers, we will provide a detailed summary and explanation of the construct meaning for each signal in the following text. All the signals and related studies are shown in Table 4.

**Table 4:** Signals impacting crowdfunding performance in RECF and studies.

Signals Category	Sub-signals impacting crowdfunding performance	Studies
Projects-related	goal	[4, 5, 8, 29, 30, 34, 39]
	duration	[4, 5, 8, 40]
	title-, text-or description-related	[4, 5, 7, 8, 12, 26, 29, 31–33, 35–37, 39, 41]
	reward-related	[5, 10, 28, 35, 39, 41]
	video, image	[4, 5, 7–10, 12, 13, 37, 39, 40]
	preparation time	[5]
	FAQ	[5]
	product quality	[30]
	funding model (KIA or AON)	[8]
	patent	[37]
	popularity	[5]
Fundraisers-related	biography	[29]
	experience as fundraisers	[4, 7, 13, 39, 40]
	experience as backers	[40]
	update	[5, 6, 8, 10, 34, 38, 39]
	reply	[6, 13, 40]
	self-pledge	[12]
	social network, Facebook friends	[5, 39]
	credibility	[41]
	integrity	[41]
	team-related	[8]
Backers-related	comments	[6, 9, 10, 13, 34, 35, 38, 40, 41]
	Facebook sharing, Facebook comments, Facebook likes	[5, 8, 38, 39]
	prior funding	[6]
	prior comments	[6]
	Backer’s support	[26]
	External media or consumer	[41]

#### Projects-related signals

Projects-related signals refer to the characteristics or attributes associated with the project itself. The most common signals are based on project descriptions, project titles, or text-related signals, such as length, narrative styles, tone, subjectivity or objectivity, language style, entrepreneurial orientation, etc. Kunz et al. [5] take the word count of description as a measure of project preparedness and found that it is positively associated with crowdfunding performance. Zhao et al. [12] conducted empirical research and demonstrated that the effect of the fundraiser’s self-funding on crowdfunding performance is mediated by project description quality. In exploring different textual strategies for project descriptions, Wang et al. [29] discuss the impact of subjectivity and objectivity. The author proposes that for a detailed textual description, objective content should be positioned at the beginning of the narrative, followed by subjective statements, to enhance the success of online fundraising.

In addition to project descriptions, the media usage of the project, including video and image-related content, has also received a lot of attention. Li et al. [10] believe that videos and images are signals of quality. They found that the quantity of images plays a significant and positive role in the successful funding of the project. Although video isn’t statistically significant, it positively influences the likelihood of successful financing. Courtney et al. [9] suggest that video and image signals can alleviate concerns about information asymmetry regarding project quality and fundraiser credibility, thereby increasing the possibility of crowdfunding performance. Some studies explored the quality of video and found that video quality has a positive effect on crowdfunding performance by shaping impressions held by potential funders [37]. Besides, video quality can positively moderate the impact of costly signals on crowdfunding performance.

Some scholars have also explored reward-related signals in their research. From the information economics perspective, Sewaid et al. [28] examined the impact of reward pricing commitment and the publicized discount on crowdfunding. They uncovered that signaling about the future retail price positively affects project performance. Some studies indicated that the number of rewards positively affects crowdfunding [5] while others suggested that the relationship is insignificant [10]. Besides, some scholars discussed the delivery time of reward [5, 35]. Ahsan et al. [41] developed a theoretical framework and proposed that the utilitarian and hedonic characteristics of rewards, along with innovativeness, influence funders’ decisions and, consequently, affect project fundraising.

Other common signals include the funding goal and the duration of the project. Studies pointed out that goal has a negative impact on crowdfunding performance, which is consistent with goal-setting theory [5, 8]. Usman et al. [34] compared the differences in the effects of project signals on fundraising between China and the UK. His findings suggest that setting higher goals correlates more positively with project success in the UK than in China. Regarding the duration of the project, different studies have reached varying conclusions. Some research has found that longer durations increase the likelihood of project success [8], while others have noted a negative effect [4, 5]. Additionally, some studies have found no significant direct impact, but an interaction effect with other signals [40].

Furthermore, the funding model, delivery time, preparation time, FAQ, patent ownership [37], and popularity are discussed. For example, all-or-nothing (AON) model projects are more likely to be funded [8]. Kunz identified "staff picked" as an indicator of a project’s popularity and argued that the presence of a project’s FAQ and preparation time are representations of project preparedness. Utilizing data from Kickstarter, Kunz explored the impact of various signals on crowdfunding performance [5].

#### Fundraiser-related signals

Fundraiser-related signals are those signals that are related to individuals who launched the projects. There are seven papers that studied the impact of updates on crowdfunding performance. The conclusions are inconsistent. Song et al. [6] found that there is no significant but positive effect of updates on fundraising performance and the effect may disappear gradually. Usman et al. [34] demonstrated that updates are negatively associated with project success, especially in China. Li [10] believes that updates influence the interactive trust process and have a positive impact on project fundraising. Another common signal is fundraiser experience, which refers to previous experience as fundraisers or entrepreneurs. Fundraiser experience serves as a signal of the fundraiser’s credibility, and the impact on performance differs between experiences of success and failure [7]. Most studies uncovered that there is a significantly positive relationship between fundraiser experience and crowdfunding performance [9, 13, 35, 40] while the moderation effect of fundraiser experience is also investigated [13, 27]. Meanwhile, other studies explored experience as backers, which refers to the number of projects that the fundraiser backed [40].

Furthermore, the reply is also a common signal in selected papers. It is viewed as fundraiser engagement [6] or communication [13] with backers that can mitigate information asymmetry in reward-based crowdfunding. They pointed out that reply has a positive impact on project success and some synergistic effects are also discussed. The social network of fundraisers (i.e. Facebook friends) can also affect project fundraising [5]. Besides, the fundraiser’s self-funding behavior is also a significant signal. Zhao et al. [12] indicated that self-funding is positively associated with project success. Moreover, this effect is partially mediated by the quality of projects’ signals. Research rarely explored biography, integrity, and team related to fundraisers. For example, from the resource-based view, projects with large entrepreneurial teams are more likely to succeed [8].

#### Backer-related signals

Some signals that reflect the characteristics of backers can also affect crowdfunding performance. The most common signal is the backer comment, which plays a role in communication and electronic word-of-mouth (eWOM) during the crowdfunding process. Existing research primarily explores the number of comments [13, 34] and the sentiment of comments [6, 9]. The results indicate that a higher number of comments positively affects crowdfunding performance, with positive comments having a beneficial effect and negative comments having a detrimental impact. In addition to this, the social media activities of funders have also received extensive attention, primarily including Facebook likes [39], Facebook sharing [8], and Facebook comments [5]. These studies all highlight the importance of interactions among funders for the success of project fundraising. Finally, a small number of studies have noted the herding effect among funders, specifically how prior funding and prior comments influence subsequent contributions [6]. Additionally, some niche research conceptually explored the impact of external media or consumer signals on the fundraising of reward-based crowdfunding projects [41].

In conclusion, the signals influencing the success of crowdfunding campaigns have complex interactions. Therefore, analyzing signals affecting crowdfunding performance solely using regression models is insufficient. Besides, crowdfunding platforms are typically two-sided platforms, with the supply side being the fundraisers and the demand side being the investors. There are complex cross-side network effects between fundraisers and backers. Therefore, the fundraising performance of crowdfunding projects is influenced by these signals together, rather than by the isolated effect of any single factor. Additionally, signaling theory has called for papers to investigate the combined effect of signals [112]. A comprehensive understanding of different signal combinations and the substitution effect or compensatory effect among them remains to be uncovered.

To address this research gap, based on signal theory and using the fsQCA approach and linear regression from a configurational perspective, this paper intends to explore the combined impact of different signals from three aspects: fundraiser engagement, third-party endorsement, and project preparedness. It aims to reveal the various patterns of high fundraising performance in crowdfunding projects.

## Theoretical framework and hypothesis development

### Signal of fundraiser engagement

According to the homepage of the fundraiser on the Indiegogo crowdfunding platform, engagement is the name of the game when it comes to crowdfunding. Sharing thoughts on campaigns can help fundraisers and strengthen the Indiegogo community [113]. Fundraiser engagement can significantly impact crowdfunding performance for several reasons. First, it can build trust and credibility. Active engagement from the fundraiser builds trust and credibility with potential backers. When fundraisers provide updates and engage promptly with the community, backers are more likely to believe in the project’s legitimacy [1, 9, 114]. Second, it helps with transparency and communication. Open and transparent communication is crucial in crowdfunding. Fundraisers who actively update progress, challenges, and changes in plans maintain transparency, which helps backers feel more involved and informed [114]. Third, fundraiser engagement can strengthen the community. Engaging with backers fosters a sense of community around the project. This community can become a powerful support network, helping to spread the word about the campaign and encouraging others to participate [49]. Last but not least, it can improve backer satisfaction. Engaged fundraisers tend to keep backers satisfied by addressing concerns, providing timely updates, and involving backers in the project’s development [1]. Satisfied backers are more likely to support future projects from the same fundraiser.

### Signal of third-party endorsement

In the context of crowdfunding or marketing in general, a third-party endorsement is an external source that provides approval, support, or recommendation about a product or project [9, 115]. it can impact the campaign’s success in several ways. First, third-party endorsement may play the role of peer influence, especially when it comes to backer comments or social media sharing. Third-party endorsements can also help alleviate the information gap concerning start-up legitimacy. Feedback from backers who have supported the campaign provides social proof [60, 93], contributing to the overall trustworthiness of the project in the eyes of potential backers. When backers publicly endorse a project on social media [19], it provides social validation. Other individuals may be more inclined to support the campaign when they see that it is gaining positive attention and support from real people. Second, third-party endorsement may act as word-of-mouth marketing [114]. Friends and connections of backers are likely to take notice, and recommendations from people within their social circles can carry significant influence. Third, third-party endorsement can also be beneficial for building a crowdfunding community [1, 60, 116]. A thriving community can foster a sense of belonging and encourage others to join in, creating a positive feedback loop that enhances the campaign’s success. Interaction between backers and potential backers in the comments section can address questions, provide additional information, and build a sense of community involvement, all of which are conducive to crowdfunding performance.

### Signal of project preparedness

A crowdfunding project’s videos, images and description play a crucial role in project preparedness and can significantly impact crowdfunding performance for several reasons. The video or images and description are powerful tools for showing project quality [13, 117]. Under the high level of information asymmetry, backers often have limited information regarding the fundraiser’s credibility and their actual ability to produce and deliver the promised product or service. Therefore, the well-prepared videos or images and description can provide detailed information to potential backers. They can perceive reliability from adequate information shown in project pitch. Besides, well-prepared projects can express the overall professionalism of the crowdfunding fundraiser [1]. This professionalism helps build trust with potential backers, assuring them that the project is well-planned and that their contributions will be used responsibly. More importantly, project preparedness is an effective tool to mitigate information asymmetry in the crowdfunding context, which is essential to crowdfunding performance [9, 13].

### Trust from signals

Due to the information asymmetries underlying online transactions, the gaining of trust in online marketplaces has been widely recognized as one of the prime objectives in crowdfunding. Earlier research has investigated the role of trust in crowdfunding performance. Different studies have explored the impact of various types of trust on project financing. For example, Lui et al. [94] investigated the influence of webpage cues on crowdfunding performance from the perspectives of competence trust and benevolence trust. Competence trust refers to the dependability, reliability, and ability of a partner to perform their required role in an agreement. Competence trust mainly comes from the project quality, the ability of fundraisers, and third-party endorsement. Benevolence trust lessens relational risks related to the goodwill and reciprocal care of entrepreneurs as a partner in uncertain situations. Backers will perceive benevolence trust when fundraisers are willing to expose some offline information and post frequent updates to share more information.

Other studies [14, 79, 118] explained the effects of calculus trust and relationship trust on crowdfunding financing. Calculus trust is based on economic calculation and is an ongoing economic calculation whose value is derived by comparing the outcomes resulting from creating and sustaining the relationship to the costs of maintaining or severing it. Relationship trust refers to trust that is based on repeated interaction between individuals that involves care and concern, which has a more relational orientation. Different types of trust originate from different signals, and these trusts are not entirely independent but rather have an interplay. For example, calculus trust serves as the foundation for relationship trust and can, to some extent, be transformed into relationship trust [14]. We will utilize those two trusts classification in our subsequent hypothesis development.

### Interplay between signals

#### Linkage between signals

The interplay effect between signals has two main sources: the inherent interaction between signals and the mutual influence between the different trust generated by different signals. We first discuss the inherent interaction between signals. On the one hand, the interplay between fundraiser engagement, third-party endorsement, and project preparedness can have a synergistic effect on crowdfunding performance. When these elements work together cohesively, they enhance various aspects of a crowdfunding campaign, increasing the likelihood of achieving funding goals. For example, fundraiser engagement fosters a direct connection with backers, demonstrating commitment and responsiveness. When combined with third-party endorsement, which adds external validation, and project preparedness, which showcases planning and transparency, the overall trust and credibility of the campaign are significantly enhanced. On the other hand, these signals may substitute or compete with each other and generate an inhibiting interaction effect. In some cases, a project might overly rely on third-party endorsements to validate its credibility, neglecting direct engagement with the community members. This can create a situation where backers feel disconnected from the fundraiser and may question the legitimacy of the project. Backers may question the fundraiser’s motivations and whether the project is genuinely driven by passion and commitment.

Second, potential backers perceive trust differently based on various signals. Some signals generate calculus trust (trust from head). Calculus trust is created from the evaluation of conditions and information weighing costs and benefits, such as those signals related to project quality, project videos, and third-party endorsements. Other signals can help potential backers establish relationship trust (trust from heart), which arises from emotional bonds and social identification between the parties, such as social ties or frequent updates from the project fundraiser. Both types of trust help to alleviate information asymmetry, thereby enhancing the performance of project fundraising.

However, there is an interaction relationship between calculus trust and relationship trust. Specifically, calculus trust may also serve as a foundation for relationship trust [14]. In crowdfunding scenarios, a higher degree of calculus trust diminishes backers’ uncertainties about a fundraiser’s dependability. This form of trust emerges from an initial rational evaluation, helping backers decide whether to trust a fundraiser, thereby enhancing the fundraiser’s credibility and fostering a high-quality interaction between the two parties. Consequently, this foundational trust encourages backers to form stronger emotional connections with the fundraiser. Therefore, calculus trust acts as a prerequisite for the development of relationship trust. Emotional bonds, indicative of relationship trust, only begin to form once a fundamental level of calculus trust is established. Over time, this calculus trust can evolve into relationship trust.

As previously stated, signals from project fundraisers, backers, and the project itself can all influence crowdfunding performance. These signals should not act independently but interact together to impact fundraising performance. The interaction among these signals has been to some extent confirmed [7, 13]. For example, some research has proved that backer comments complement or substitute for the effects of signals originating from the campaign or fundraiser [13]. Consistent with prior research, this paper posits that crowdfunding platforms are typical two-sided platforms, with fundraisers and backers being stakeholders with complementary interactions on the crowdfunding platform [43]. As the entity that connects both sides, the attributes of the project itself also influence their relationship. Furthermore, the fundraising for a project commonly lasts over a while, usually 30, 45, or 60 days. Crucial signals undergo changes during distinct phases of the fundraising process [19, 119]. To achieve ultimate fundraising success, a project needs to rely on various signals. Therefore, this paper contends that achieving high project performance necessitates the combined effect of various signals and cannot solely rely on a single type of signal. We thus develop the following propositions:

**Proposition 1:** To achieve high crowdfunding performance, different signals need to work together, single factor is not sufficient to produce high fundraising performance.

#### The role of project preparedness

Reward-based crowdfunding begins with the innovative idea of the project fundraiser. Most projects are only in the conceptual and creative stage [120], leading to high uncertainty and significant information asymmetry in crowdfunding [7]. Backers face inherent risks when supporting a crowdfunding project. Thus, backers need diverse signals to make their decisions [13]. Project preparedness may offer detailed information, but it might not effectively communicate the passion and commitment of the fundraiser. A well-prepared project might lack visibility without active fundraiser engagement and third-party endorsements.

The crowdfunding market is highly competitive, with numerous projects struggling for attention simultaneously [43]. In a competitive crowdfunding environment, projects need to stand out. Merely having a prepared project might not be enough to stand out among the diverse range of campaigns competing for backers’ contributions. Project preparedness may focus heavily on providing information about the project but may not effectively convey the passion, dedication, or personal story of the fundraiser. Therefore, potential backers mainly perceive calculus trust from project preparedness, but lack relationship trust, which is crucial for enhancing their willingness to invest.

Crowdfunding campaigns are dynamic, and challenges can arise unexpectedly. Project preparedness, often presented through text, images, and videos, may be static. Without dynamic elements or ongoing updates, it might not effectively capture the evolving interest of potential backers throughout the campaign. A well-prepared project may not effectively leverage a feedback loop with backers. Continuous interaction and feedback integration can enhance the project dynamically [51, 114], addressing backers’ concerns and adapting to their preferences throughout the campaign. We thus develop the following propositions:

**Proposition 2:** Project preparedness should work with fundraiser engagement and third-party endorsement, rather than work alone to produce high performance.

#### Fundraiser engagement-driven pattern

In different trust conditions, the interplay of signals, namely different strategies can yield varying outcomes. According to previous studies [118], there are four crowdfunding marketing strategies tailored to different trust conditions. The minimalist strategy is effective in scenarios with an overall trust surplus, relying on minimal investments in content and social media to leverage existing high trust among potential backers for securing funding. In contrast, the technician strategy is tailored for conditions marked by an informational-trust deficit; it requires substantial investments in quality content to build calculus trust, focusing on addressing backers’ detailed concerns while minimizing social media engagement. Meanwhile, the influencer strategy is designed for situations with a relational trust deficit, emphasizing extensive social media engagements to enhance relational trust through social proof and third-party endorsements, thereby deepening connections with backers. Lastly, the innovator strategy targets campaigns facing an overall trust deficit, necessitating heavy investments in both quality content and social media engagements to comprehensively enhance both calculus and relational trust, aiming for overall campaign success.

Some fundraisers are not professional entrepreneurs and may not be good at crafting business plans and storytelling. The videos and project descriptions of these projects may not stand out enough. In this context, fundraiser engagement and third-party endorsement can compensate for this shortcoming. Fundraiser engagement, specifically through fundraiser comments, plays a pivotal role in fostering a sense of community and establishing social connections with backers. The motivation of backers is not only driven by economic incentives such as receiving rewards [53, 71] but also by emotional motives, such as altruism [105]. Therefore, establishing an emotional connection with backers can reduce perceived uncertainty and influence their contribution decisions. Additionally, updates can provide additional project information which increases transparency and enhances the project’s legitimacy [121]. In conclusion, frequent updates can generate calculus trust by answering backers’ concerns with detailed information. Fundraiser comments can be a source of relational trust to maintain a high level of connection with backers. Hence, fundraiser engagement can serve as a key element, driving high crowdfunding performance even in situations where project preparedness is relatively weak, which is similar to the technician strategy. We thus develop the following propositions:

**Proposition 3:** When project preparedness is insufficient, fundraiser engagement acts as a complement, leading the project to achieve high fundraising performance (fundraiser engagement-driven pattern).

#### Third-party endorsement-driven pattern

Similarly, strong third-party endorsements can compensate for inadequate project preparedness. They play a pivotal role in addressing the legitimacy of a startup [90, 122]. First, in the crowdfunding context, a distinctive form of third-party endorsement emerges through a collective set of backer comments [115]. It is common for backers to share their thoughts about a project on the crowdfunding platform. Backer comments function as information intermediaries, conveying and evaluating information available about the fundraiser and the projects. Information conveyed through third-party endorsements helps backers evaluate both project quality and founder credibility. Therefore, it can bring more calculus trust by providing more information. Second, backer comments indicate a heightened expectation regarding the project’s worthiness, providing valuable insights for potential backers to evaluate the project’s quality or validate their assessments before deciding to support the project [90]. Backer perceptions and opinions serve as peer evaluations that significantly influence others’ viewpoints [123, 124]. Third, except for backer comments, Facebook sharing is the other common form of third-party endorsement [19]. Facebook sharing by backers can extend the reach of the crowdfunding campaign beyond the immediate network of the project fundraiser. Each share has the potential to reach a broader audience, increasing visibility and attracting new backers who may be interested in the project [21]. Fourth, Facebook sharing also builds social interaction between parties, which may enhance relational trust. Therefore, similar to the innovator strategy and influence strategy, adequate third-party endorsements compensate for the lack of project preparedness, driving crowdfunding projects to achieve high fundraising performance. We develop the following propositions:

**Proposition 4:** When project preparedness is insufficient, third-party endorsement acts as a complement, leading the project to achieve high fundraising performance (third-party endorsement-driven pattern).

**Fig 2:**
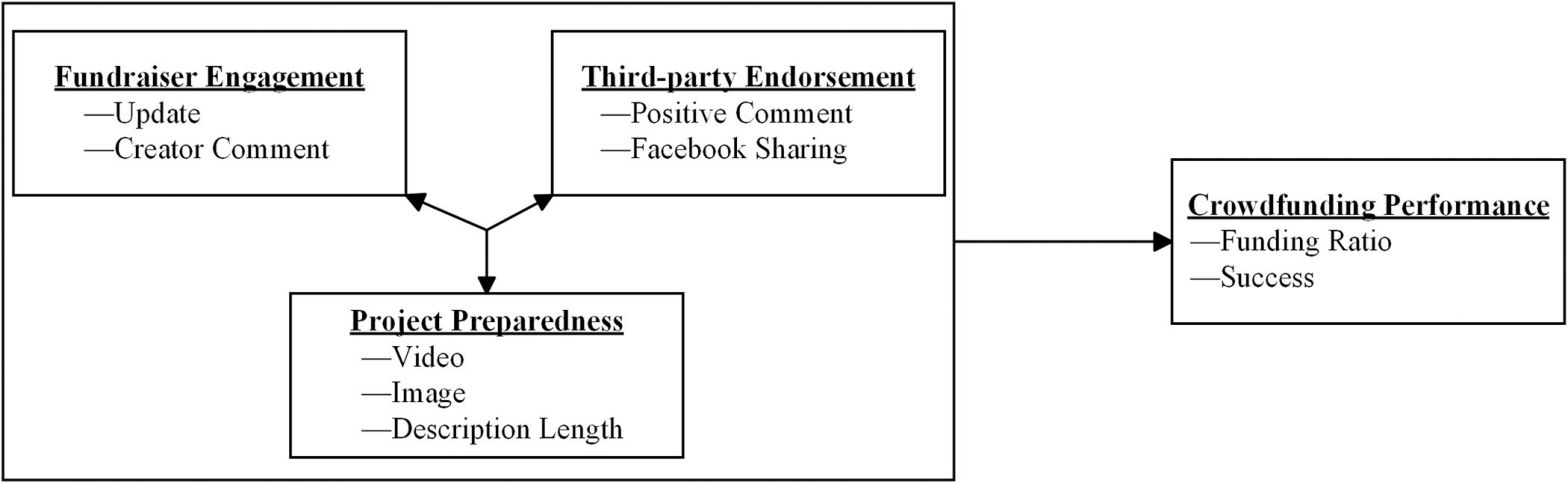
Nomological network of configurations producing crowdfunding performance.

Fig 2 depicts the nomological network of our research. This model illustrates the configuration paradigm used to build a configuration theory that explains the complex simultaneous interactions among fundraiser engagement, third-party endorsement, project preparedness, and crowdfunding performance.

### Data and variables

We used a Python-based web data extraction method to capture crowdfunding project data from the Indiegogo crowdfunding platform (http://www.indiegogo.com). Indiegogo is the largest and most dominant reward-based crowdfunding platform and has been widely used in existing crowdfunding research. Our dataset contains both two types of funding models (keep-it-all and all-or-nothing). Our final sample is comprised of 6,284 projects of which 5,124 failed and 1,160 were successful. These projects received pledges totaling 23,757,721 dollars from 268,280 backers. The 5,124 failed projects gained pledges of 1687 dollars on average, compared to pledges averaging 13026 dollars from the 1160 backers. The final dataset contains three major categories and 28 sub-categories: Tech & Innovation (e.g., Audio, Camera Gear, Education, Energy & Green Tech, Fashion & Wearables, Food & Beverages, Health & Fitness, Home, Phone & Accessories, Productivity, Transportation, and Travel & Outdoors), Creative Works (e.g., Art, Comics, Dance & Theater, Film, Music, Photography, Podcasts, Blogs & Vlogs, Tabletop Games, Video Games, Web Series & TV Shows, and Writing & Publishing), and Community Projects (e.g., Culture, Environment, Human Rights, Local Business, and Wellness).

#### Performance measurement

Following previous studies [13], we choose funding ratio as a measurement of crowdfunding performance, which equals the proportion of project fundraising amount to the fundraising goal. When calculating the funding ratio, only the contributions made during the fundraising period were included to more accurately and equally reflect the project’s fundraising outcome. Although some research considers crowdfunding success as the outcome variable, measuring performance by the funding ratio, a continuous variable allows for a more detailed representation of the project’s fundraising performance level. Meanwhile, we use crowdfunding performance to measure crowdfunding performance as one robustness check.

#### Fundraiser engagement

We choose updates [49] and fundraiser comments [113] as the measurement of fundraiser engagement, which are the main ways for fundraisers to engage in the crowdfunding community. After initiating a project on the crowdfunding platform, the fundraiser can post project updates in the provided update section on the platform, providing backers with information about the project’s progress, plans, and more. In addition, project fundraisers can engage with the crowdfunding community by posting comments, sharing their perspectives and insights, and interacting with other stakeholders. The Indiegogo platform refers to this behavior as engagement. Besides, updates and fundraiser comments are used in previous studies and have been confirmed as determinants of crowdfunding performance [1, 49]. Therefore, we choose those two variables (update and fundraiser comment) as a measure of fundraiser engagement. For fundraiser comments, the measurement rule is based on whether the commenter is the same as the fundraiser or a member of the fundraiser’s team. If they are the same, it is counted as a fundraiser comment.

#### Third-party endorsement

We choose two variables as third-party endorsements, which have been verified as valid signals that affect crowdfunding performance [13]. The one is the number of positive backer comments [9, 115]. Backer comments, acting as word-of-mouth [125], represent the assessments and viewpoints of backers toward the project. Similar to consumer reviews, they provide an objective evaluation from a third-party perspective. We utilized the VADER model from NLTK to perform sentiment analysis on the comment content of each project, determining the polarity of each comment (positive, neutral, or negative). The VADER model is a simple rule-based model for general sentiment analysis, and its accuracy outperforms individual human raters (F1 Classification Accuracy = 0.96 and 0.84, respectively). After obtaining the sentiment analysis scores for each comment, we aggregated the data by project to determine the number of positive sentiment comments for each project. Thus, following past studies [9], positive comments can be viewed as third-party endorsements inside the crowdfunding platform. In addition, crowdfunding platforms offer social media sharing features, allowing backers to choose to share projects on platforms like Facebook. This helps projects attract more traffic [19]. Therefore, Facebook sharing is chosen as the other variable representing external third-party endorsement. We inputted project URLs into Facebook API (application programming interface) to retrieve the number of people who have shared the project as the measure of Facebook sharing.

#### Project preparedness

Following prior studies, we use K-means based on three variables (video, image, and project description) to measure project preparedness. Specifically, videos and images are project-originating signals and serve as a way to showcase projects, reflecting the level of effort put into developing the project and serving as an indicator of its preparedness. The project description, on the other hand, conveys essential information and details, serving as a focal point for the fundraiser’s business plan. Therefore, it also reflects the level of preparedness for the project. We conducted a K-means cluster analysis based on the number of videos, the number of images, and the word count in the project descriptions. Then we categorized the projects into two groups: high preparedness (coded as 1) and low preparedness (coded as 0).

The measures of the determinants are shown in Table 5. Descriptive statistics of the measurements are shown in Table 6.

**Table 5:** Variables and definition.

Variables	Definition
*Performance Measurement*	
Funding Ratio	The ratio of project funding to the project goal
*Fundraiser Engagement*	
Update	The number of updates during the fundraising
Fundraiser Comment	The number of comments from fundraiser during the fundraising
*Third-party Endorsement*	
Positive Comment	The number of positive comments from backers during the fundraising
Facebook Sharing	The number of Facebook “shares” of the project
*Project Preparedness*	
K-means (Video, image and description)	K-means cluster based on the number of videos, the number of images, and the word count in the project descriptions into two groups: high preparedness (coded as 1) and low preparedness (coded as 0).

**Table 6:** Descriptive statistics.

Variables	Mean	Std.dev.	Minimum	Maximum
Funding Ratio	0.4188386	0.6713539	0	14.184
K-means	0.2829408	0.4504638	0	1
Update	3.042171	7.205647	0	119
Fundraiser Comment	4.488222	23.22481	0	503
Positive Comment	4.798854	15.95833	0	649
Facebook Sharing	255.863	1139.083	0	53977

## Regression analysis

### Multicollinearity analysis

To reduce kurtosis and skewness, we used the logarithm values of description length and Facebook sharing. We tested multicollinearity between the variables. Table 7 shows that the correlations between variables are below 0.5 and far below 0.7, suggesting that multicollinearity is not a serious problem. In addition, the variance inflated factor for each independent variable is smaller than 3, suggesting the absence of multicollinearity.

**Table 7:** Correlation with variables.

	(1)	(2)	(3)	(4)	(5)	(6)	VIF
Funding Ratio (1)	1						
K-means (2)	0.0324	1					1.06
Update (3)	0.2944	0.1736	1				1.34
Fundraiser Comment (4)	0.2333	0.0941	0.4320	1			1.32
Positive Comment (5)	0.2749	0.0495	0.3289	0.3568	1		1.21
Facebook Sharing (6)	0.1791	0.1385	0.2324	0.0782	0.0477	1	1.06

### Linear regression

Following existing research on the determinants of crowdfunding performance, this paper first adopts OLS linear regression analysis to verify the significant impact of fundraiser engagement, third-party endorsement, and project preparedness on crowdfunding performance. Table 8 shows the regression results. In Model 1, we enter all the determinants we focus on. In Model 2, we add control variables (goal and duration) to ensure the validity and robustness of the results. The results indicate that preparedness (-0.0409, p = .02) is negatively associated with crowdfunding performance. Other determinants including update (.116, p = .000), fundraiser comment (.0595, p = .000), positive comment (.119, p = .000) and Facebook sharing (.0404, p = 0.000) are positively associated with crowdfunding performance. The main results provide preliminary evidence that fundraiser engagement, third-party endorsement, and project preparedness all matter in the crowdfunding process.

**Table 8:** Linear regression results.

	(1)	(2)
	Funding Ratio	Funding Ratio
*Project Preparedness*		
K-means	-.0485**(.0178)	-.0409*(.0176)
*Fundraiser Engagement*		
Update	.117***(.0092)	.116***(.0091)
Fundraiser Comment	.0574***(.0089)	.0595***(.0088)
*Third-party Endorsement*		
Positive Comment	.119***(.0083)	.119***(.0083)
Facebook Sharing	.0385***(.0036)	.0404***(.0036)
constant	.0405*(.0172)	1.48***(.0652)
controls	NO	YES
*N*	6284	6284
R-squared	0.1431	0.1593
F	209.75	169.90

Note: Standard errors in parentheses

* *p* < 0.05

** *p* < 0.01

*** *p* < 0.001

More importantly, the impacts of fundraiser engagement, third-party endorsement, and project preparedness on crowdfunding financing are different, with some having a positive impact and others a negative impact. This complex impact has sparked the next step of research interest in this paper. Furthermore, this paper employs the fsQCA method to explore the interactions between different signals, including whether there is a substitution effect, offsetting effect, or compensatory effect among them. Based on the results of linear regression, this paper aims to explore different patterns of high performance in crowdfunding financing.

## fsQCA analysis

The methodology utilized in this study is Qualitative Comparative Analysis (QCA), a framework originally introduced by Ragin to address the intricacies of causal complexity within the realm of sociology [126]. Rooted in set theory and Boolean calculation, QCA delves into how combinations of antecedent conditions contribute to observable changes in outcomes [127, 128]. Unlike approaches that focus on individual elements in isolation, studies employing QCA often emphasize the interactional elements where outcomes are better explained by considering their simultaneous combinations [129].

In this study, we opt for QCA to align with a configurational approach, specifically utilizing fsQCA. The choice of fsQCA allows for the measurement of the set membership of a case as a continuous value ranging from 0 to 1. This is in contrast to other QCA variants, such as csQCA, which classifies cases into binary outcomes, or those with three or four categories, like mvQCA [126]. By employing fsQCA, we aim to more comprehensively capture the nuanced effects of changes in antecedent conditions at various levels or degrees. This fsQCA framework allows for a deeper understanding of the subtle outcomes arising from changes in causal conditions at various levels. Consequently, it precisely captures the distinctive characteristics and diversity inherent in the case [130].

FsQCA can identify different ways to achieve an outcome, showing that crowdfunding performance can be reached through various paths in different situations. By analyzing configurations, we can see the combined effects of signals on crowdfunding performance. Besides, FsQCA also helps us figure out which elements are necessary or sufficient for the outcome, providing a deeper understanding of how signals impact fundraising outcomes [7, 131, 132].

To achieve a typical and representative dataset, we choose famous projects with a large amount of fundraising. We believe that crowdfunding projects that have raised a significant amount of funds are typical examples of fundraising, which indicates legitimacy and recognition. Choosing these projects as a dataset can effectively and validly show the patterns of crowdfunding fundraising. We choose projects that are in the top 1% in terms of fundraising amount and our final data set contains 64 projects whose fundraising amounts are all over 30,000 dollars, which is an appropriate sample size for the fsQCA method.

### Data calibration

The first step to conducting fsQCA analysis is calibration to assign the determinants and outcomes into set-membership scores [133]. Calibration defines the extent to which a given case has membership in the set of, for example, a high level of crowdfunding performance, wherein three anchors should be defined: full membership, full non-membership, and the crossover point, according to empirical and theoretical knowledge of the context and cases [134]. The case membership is a real number between 0 (indicating full non-membership) and 1 (full membership).

We employed the software package fsQCA 4.0 to standardize the data and carry out our analyses. All data were adjusted to fuzzy-set calibration (0.05, 0.5, 0.95) [133]. The calibration anchors for the variables are shown in Table 9.

**Table 9:** Calibration anchors for the variables.

Variables	Anchors		
	Full non-membership	Cross-over	Full membership
*Performance Measurement*			
Funding ratio	0.27	1.00	5.16
*Project Preparedness*			
k-means	0.00	/	1
*Fundraiser Engagement*			
Update	0.00	12.00	48.00
Fundraiser Comment	0.00	3.00	254.00
*Third-party Endorsement*			
Positive Comment	0.00	14.00	384.5
Facebook Sharing	0.00	2315.50	31956.25

### Necessary conditions analysis

When performing fuzzy set truth table analysis, it is crucial to scrutinize the essential conditions to prevent their exclusion by a parsimonious solution, which could potentially lead to misleading configuration results [126]. If an element’s set membership consistently equals or exceeds the set membership in the outcome, it is likely to be deemed a necessary condition [133]. To identify these conditions, we conducted necessary conditions analysis and the results are shown in Table 10. It shows that the consistency score of each causal condition is less than 0.9, which means that no element serves as a necessary condition for crowdfunding performance (funding ratio).

**Table 10:** Necessary conditions analysis.

Determinants	Consistency		Coverage	
	High Performance	Low Performance	High Performance	Low Performance
K-means	0.493512	0.444492	0.521000	0.479000
~K-means	0.506489	0.555507	0.471794	0.528206
Update	0.711219	0.566612	0.789105	0.641724
~Update	0.677086	0.813789	0.604823	0.742039
Fundraiser Comment	0.619716	0.534814	0.793363	0.698896
~Fundraiser Comment	0.764801	0.841876	0.616948	0.693233
Positive Comment	0.728143	0.653129	0.766223	0.701565
~Positive Comment	0.716397	0.782362	0.669233	0.746040
Facebook Sharing	0.592214	0.581336	0.743695	0.745202
~Facebook Sharing	0.797102	0.800056	0.650980	0.666968

### Truth table analysis

After data calibration and necessary conditions analysis, we proceeded to perform a sufficient conditions analysis by integrating all antecedent conditions into fsQCA, aiming to investigate their collective impact on crowdfunding performance. The truth table algorithm [134] was applied to identify combinations of elements that produce the outcome of interest. The truth table includes all logically possible combinations of elements, and each row corresponds to a combination. There are three types of solutions—complex, parsimonious, and intermediate through fsQCA. A complex solution includes configurations without any counterfactuals, a parsimonious solution includes configurations with both “easy” and “difficult” counterfactuals, and an intermediate solution includes configurations only with “easy” counterfactuals [135]. Elements that are part of both parsimonious and intermediate solutions are core conditions, which have a stronger relationship with the outcome. Elements that only appear in the intermediate solution but not in the parsimonious solution are peripheral conditions, which then have a weaker relationship with the outcome.

We established a truth table using a minimum case frequency benchmark (≥1) and a raw consistency benchmark (≥0.8) [133, 135]. To refine the truth table, we applied proportional reduction in inconsistency. Using this table, we conducted a standard analysis without subjective selection for the causal conditions. We then derived parsimonious, intermediate, and complex solutions. In QCA research, it’s often more practical to opt for well-documented intermediate solutions over complex ones for reporting and interpretation. Consequently, we used the intermediate solutions to identify the number of configurations and their inclusion conditions, while the parsimonious solutions helped identify core conditions for a given configuration.

### Sufficient solutions for crowdfunding performance

Following Ragin [134] we graphically depict the result of the truth table analysis using the notation system. As shown in Table 11, solutions are enumerated, resulting in five sets of solutions for achieving high crowdfunding performance. Core elements are represented by large circles, while peripheral elements are denoted by small circles. Full circles indicate the presence of a condition, while crossed-out circles indicate its absence. Full circle elements serve as enablers for the outcome, whereas crossed-out circle elements act as inhibitors [136]. Take configuration 1 for example, the presence of an update (dark circle) means full membership in a high level of update, and a high update is an enabler. The absence (⊗) of video means the full membership in a high level of video isn’t in configurations that lead to the outcome and a high level of video is an inhibitor. By displaying the outcomes graphically, we can effectively interpret and compare the configurations to understand how these elements are combined systemically to produce the outcomes.

**Table 11:** Configurations for achieving high crowdfunding performance (funding ratio).

Causal Conditions	Con1	Con2	Con3	Con4	Con5
*Fundraiser Engagement*					
Update	●	●	●	●	●
Fundraiser Comment	●	●	⊗	●	
*Third-party Endorsement*					
Facebook Sharing	⊗		●		●
Positive Comment				●	●
*Project Preparedness*					
K-means		●	⊗		⊗
Consistency	0.962982	0.903198	0.951836	0.90281	0.932759
Raw Coverage	0.474756	0.296075	0.186575	0.470747	0.187901
Unique Coverage	0.025007	0.0012945	0.0129243	0.000143	0.002147
Solution Consistency	0.906345				
Solution Coverage	0.575069				

*Note*: ● is presence of a core condition; ⊗ is absence of a core condition; blank cells indicate either presence or absence of the element; ● is presence of a peripheral condition; ⊗ is absence of a peripheral condition.

The consistency index and coverage index are key indicators used in fsQCA research. Consistency refers to the extent to which all cases share one or more given conditions that lead to the outcomes. Generally, when the consistency is over 0.8 [133], it means that more than 80% of cases meet the consistency conditions, and X denotes a sufficient condition. If the consistency is over 0.9, then X denotes a necessary condition. Coverage refers to the extent to which these given conditions (or the combinations of conditions) could explain the occurrence of outcomes. A larger coverage index indicates greater explanatory power. consistency and coverage are calculated as follows:

$$Consistency(X_{i}\leq Y_{i})=\sum \left[min(X_{i},\;Y_{i})\right]-\sum X_{i}$$ (1)

$$Coverage(X_{i}\leq Y_{i})=\sum \left[min(X_{i},\;Y_{i})\right]-\sum Y_{i}$$ (2)

Consistency and coverage were used to validate the solutions [137, 138]. Overall solution consistency measures the degree to which all configurations together consistently result in an outcome. As shown in Table 11, the overall solution consistency is 0.9063, above the acceptable level of 0.75 [126]. Raw coverage shows the proportion of cases that have membership in their respective path to the outcome and represents their empirical importance and effectiveness. The overall solution coverage is 0.5751. That is, five configurations explained 57.5% of the cases. Therefore, our fsQCA results are valid.

Configuration 1 implies that joint presence of update and fundraiser comment are sufficient to produce high crowdfunding performance when Facebook sharing is absent. Configuration 2 shows that the combinations of update, fundraiser comment and preparedness bring high fundraising outcomes. Configuration 3 suggests that the joint presence of update and Facebook sharing are sufficient to produce high crowdfunding performance when fundraiser comment and preparedness are absent. Configuration 4 implies that update, fundraiser comment in combination with positive comment generate high crowdfunding performance. Configuration 5 shows that when preparedness is absent, update, Facebook sharing and positive comment need to work together to produce high performance. In conclusion, our four propositions are supported by the results. Specifically, proposition 3 is supported by configuration 1 and configuration 2 and configuration 4, while proposition 4 is supported by configuration 5. It is obvious that there is no configuration that contains only one signal, which provide evidence for proposition 1. From the results, proposition 2 is also supported because there is no such configurations showing only the presence of project preparedness can produce high performance.

### Robustness check

We also performed several sensitivity tests to check the robustness of our results. First, we changed the consistency thresholds for sufficiency analysis from 0.8 to 0.85 [7], a more stringent level. Second, we changed the anchors for the outcome and antecedent variables in the calibration process from (0.95,0.5,0.05) to (0.9,0.5,0.05) for full membership, crossover point, and full non-membership. With this new calibration, we conducted new fsQCA analyses and found that configurations show the same patterns and structures as configurations in Table 11, except for the subtle changes in consistency and coverage of each configuration as well as the overall solution consistency and coverage.

At last, we change our outcome variable into crowdfunding success. we defined a project as successful (code as i) if it reached or exceeded its pledging goal. Otherwise, it was defined as unsuccessful (coded as 0). We then conducted a sufficiency analysis to identify conditions that are sufficient to produce high crowdfunding performance. The results are shown in Table 12. In summary, these additional tests confirm that our fsQCA results are robust.

**Table 12:** Configurations for achieving high crowdfunding performance (crowdfunding success).

Causal Conditions	Con1	Con2	Con3	Con4	Con5
*Fundraiser Engagement*					
Update	●	●	●	●	●
Fundraiser Comment	●	●	⊗	●	
*Third-party Endorsement*					
Facebook Sharing	⊗		●		●
Positive Comment				●	●
*Project Preparedness*					
K-means		●	⊗		⊗
Consistency	0.875753	0.878636	0.856637	0.877074	0.868339
Raw Coverage	0.290936	0.194085	0.113149	0.308170	0.117872
Unique Coverage	0.017063	0.004893	0.008723	0.001914	0.002511
Solution Consistency	0.879572				
Solution Coverage	0.376064				

*Note*: ● is presence of a core condition; ⊗ is absence of a core condition; blank cells indicate either presence or absence of the element; ● is presence of a peripheral condition; ⊗ is absence of a peripheral condition.

## Discussion

This study takes configurational perspectives to provide a more nuanced understanding of crowdfunding performance by exploring how fundraiser engagement, third-party endorsement, and project preparedness combine into configurations to affect crowdfunding performance. Fundraiser engagement is measured by updates and fundraiser comments, third-party endorsement is measured by positive comments and Facebook sharing, and project preparedness consists of video, image, and project description length. First, using OLS regression to clarify the significant impact of each signal on crowdfunding performance provides a solid foundation for subsequent analysis. The regression results indicate that most signals positively impact crowdfunding performance, while only the influence of preparedness is negative. Following previous studies, these results show the importance of signals on crowdfunding performance [5, 10, 139] and also suggests that the combination of signals may have a complex impact on crowdfunding performance. Then, by examining the combined effects of those signals on fundraising performance, fsQCA is used to identify multiple equifinal configurations associated with outcomes based on secondary data from reward-based crowdfunding platform Indiegogo. Finally, the links between fundraiser comments, third-party endorsement, and crowdfunding performance are theorized, and the proposed concrete configurational recipes reveal how projects can leverage fundraiser engagement, and third-party endorsement to achieve high crowdfunding performance.

### Theoretical implications

This paper offers several important theoretical contributions to crowdfunding literature. First, we extend the determinants of crowdfunding performance by exploring the interplay of multiple signals on crowdfunding fundraising performance [9, 13, 17]. Exploring combinations of signals is particularly relevant in high information asymmetry environments such as crowdfunding where multiple signals are needed to make the right decision. Instead of only adopting a traditional correlation-based approach that examines the linear net effects, we shed light on the combined effect of different signals related to fundraisers, backers, and projects by adopting a configurational method with fsQCA. Our OLS regression results first confirm the importance of those signals, which are consistent with previous literature [5, 10]. Furthermore, the fsQCA results reveal that signals need to work together to produce crowdfunding performance, rather than operating solely from each other. Our contribution is important as it addressed the calls for “simultaneous interactions of multiple signals [15]. We uncovered that different signals can complement each other and certain signals (fundraiser engagement and third-party endorsement) can substitute the absence of others (project preparedness). Besides, we summarized them into two patterns that prove different patterns to generate high crowdfunding performance. With the configurational perspective, our work indicates that diverse sets of signals, each conveying unique information, can result in positive outcomes. Additionally, our study also demonstrates the important role of two types of trust (calculus trust and relationship trust) in crowdfunding. One type of trust alone is not sufficient for crowdfunding projects; both types of trust need to work together to help the project succeed.

Second, we add to the body of knowledge concerning the influence of social media activities on crowdfunding performance [19, 140]. In contrast to studies that predominantly emphasize the positive impact of social media, this research discovers that, even in the absence of social media activities (Facebook sharing), high fundraising performance can still be achieved by enhancing fundraiser engagement and project preparedness. This finding holds particular significance for fundraisers with limited social capital who are not good at attracting external traffic to their projects. Our results reveal that frequent project updates, and active community engagement by the fundraiser, coupled with effective storytelling, can also enhance backer satisfaction, increase the project’s legitimacy, and foster trust among backers, thereby assisting the project in achieving crowdfunding performance.

Third, we provided a comprehensive literature review on the signals influencing the performance of reward-based crowdfunding, with a particular emphasis on research based on signaling theory. There is a substantial body of research on signals influencing crowdfunding performance. After conducting a comprehensive literature review and analysis, this paper describes the basic characteristics of the studies (such as publication year and quantity) and the theoretical foundations employed. The studies that use signal theory to investigate the determinants of reward-based crowdfunding are further analyzed. The signals are summarized from three perspectives: fundraisers, backers, and project attributes. This literature review helps the researcher comprehend the findings of previous empirical studies, which will be useful for future studies.

### Practical implications

This study offers some practical suggestions for fundraisers on how to leverage their capability to achieve contributions. First, the fundraiser needs to pay attention to leverage all aspects of signals. According to our research results, the success of crowdfunding projects does not solely depend on a single factor but is the result of the combined effects of multiple signals. Fundraisers need to be aware of this during the project fundraising process to help the project achieve a higher funding amount. Second, for fundraisers with low social capital, such as those with a small number of Facebook friends, during the project fundraising period, fundraisers need to actively participate in the crowdfunding community, maintain contact with backers, and pay attention to updating project progress. This helps compensate for the absence of third-party endorsement through fundraiser engagement. Third, novice fundraisers who are not good at storytelling, during the project fundraising process, can enhance the legitimacy of the project by publishing project updates (fundraiser engagement) and increasing project social media activities (third-party endorsement). This compensates for the absence of project preparedness and helps the project achieve fundraising success.

### Limitations and future research directions

Our work has several limitations and future directions to study. First, our fsQCA results may show several paths that produce high crowdfunding performance, but ignore many other potential contextual variables, such as project perceived risk and different project categories. Future research is needed to make our theory and model more generalizable by exploring effective solutions under different contexts.

Second, the variable combinations in this article could be more precise and comprehensive. For example, fundraiser engagement could further focus on variables related to the fundraiser’s responses or contributions, and the sentiment of backer comments could be selected as a measure of third-party endorsement. This would allow for a more complete and detailed exploration of the impact of these signals on crowdfunding fundraising.

Third, certain constraints on the applicability of our solutions also inform our recommendations for future research. Since we only collect data from the Indiegogo platform, the generalizability of our findings is limited by the particular operation model of this single platform. Future studies could be conducted using data from different platforms or data from different time windows to generate more significant findings.

## Supporting information

S1 Table Selected studies on signaling in reward-based crowdfunding. (DOCX)

S2 Table Truth table for high crowdfunding performance (funding ratio). (DOCX)

S3 Table Truth table for high crowdfunding performance (success). (DOCX)

S1 Dataset (XLS)
