# Supplementary material for: Fundraiser engagement, third-party endorsement and crowdfunding performance: A configurational theory approach
Source: PLoS One. 2024 Aug 15;19(8):e0308717. doi: 10.1371/journal.pone.0308717 (PMC11326654; doi:10.1371/journal.pone.0308717)
Supplement: S1 Table — (DOCX) [file pone.0308717.s001.docx]

**S1 Table. Selected Studies on Signaling in Reward-based Crowdfunding**

| **ID** | **Year** | **Journal** | **Title** | **Findings** |
| --- | --- | --- | --- | --- |
| 1 | 2024 | Journal of the Academy of Marketing Science | The time-varying effects of rhetorical signals in crowdfunding campaigns | We demonstrate that (a) the four rhetorical signals are positively related to funding formation, (b) emotional and cognitive tone exert stronger effects in early phases of projects, whereas linguistic style match and communal language style exert stronger effects in later phases, and (c) the dynamic effects of rhetorical signals on funding formation are greater when the number of backers increases. |
| 2 | 2022 | Small Business Economics | Signalling entrepreneurs’ credibility and project quality for crowdfunding performance cases from the Kickstarter and Indiegogo environments | identify two distinct signal- ling patterns that show entrepreneur’s credibility and project quality signals can complement each other to produce crowdfunding performance. In an environment with less uncertainty, entrepreneur’s credibility in terms of crowdfunding experience can also compensate absent project quality to produce crowdfunding performance. In an environment with higher uncertainty, entrepreneur’s credibility and project quality need to be both present to establish the necessary legitimacy for crowdfunding to be successful. Furthermore, by integrating positive (i.e. success) and negative (i.e. failure) signals, we demonstrate how signal incongruence can enhance crowdfunding performance. |
| 3 | 2021 | Small Business Economics | “Pledge” me your ears! The role of narratives and narrator experience in explaining crowdfunding performance | Our findings reveal that greater success in collecting funds is achieved by RBCF campaigns that are communicated through RIP narrative styles rather than OJ styles. showing that entrepreneurs with extensive experience in RBCF attract more pledges by adopting OJ narratives rather than RIP narratives. |
| 4 | 2021 | Journal of the Association for Information Systems | Mitigating Information Asymmetry to Achieve Crowdfunding performance: Signaling and Online Communication | we propose that signals from campaigns (videos) and fundraisers (experience) can mitigate information asymmetry concerns about project quality and fundraiser credibility and that fundraiser-originating signals offset the effect of campaign-originating signals on crowdfunding performance. Further, we posit that online communication between fundraisers and backers (backer comments and fundraiser replies) complements or substitutes for the effects of signals originating from the campaign or fundraiser. |
| 5 | 2021 | Research Policy | Resolving information asymmetries in financing new product development: The case of reward-based crowdfunding | We find that signaling information regarding the future retail price enhances campaign performance. Moreover, when the different signals originate from the same source and overlap in the information they convey, the more informative signals partially offset the effect of the less informative signals. |
| 6 | 2021 | Computers in Human Behavior | Signaling persuasion in crowdfunding entrepreneurial narratives: The subjectivity vs objectivity debate | We observe a statistical difference between the text sections in a narrative. Some level of objective appeal should reside in the abstract and the reward statement. To court investors, the title, detailed textual description, and biography should be phrased subjectively. For the detailed textual description, the objective contents should be positioned at the start of the narrative, followed by the subjective statements, to enhance the success of online fundraising. Further, the overall prediction accuracy of funding support in the presence of subjective versus objective signals improves by 1.76%, from 76.37%, as shown through machine learning prediction analytics. |
| 7 | 2021 | Manufacturing & Service Operations Management | Signaling to the Crowd Private Quality Information and Rewards-Based Crowdfunding | We find that the entrepreneur should signal high quality by setting a high target that is distorted above the full information optimal level. While a separating equilibrium always exists, a pooling equilibrium can only occur under very specific circumstances. We show that the high target affects the quality choice of entrepreneurs and may deter unique, high-quality projects. In addition, we discuss how the entrepreneur should modify the signaling strategy when a high target potentially deters backers from pledging because of the cost of participating in a failed campaign. |
| 8 | 2021 | Business Horizons | Skin in the game: Self-funding and reward crowdfunding performance | Our results demonstrate that entrepreneurs’ direct self-funding is positively associated with crowdfunding performance. Moreover, this effect is partially mediated by the quality of campaigns’ content elements. |
| 9 | 2021 | Journal of Business Research | The effects of brand prominence and narrative features on crowdfunding performance for entrepreneurial aftermarket enterprises | Analysis of data from 343 crowdfunding projects shows the positive influence of brand prominence, language style, and narrative length on funding success. Findings also show that brand prominence increases the effects of language styles and narrative length on funding success. |
| 10 | 2020 | Information Technology & People | Exploring the dynamic influences and interaction effects of signals on backers’ investment in the crowdfunding market | The findings demonstrate that the work of different signals is significantly effective at the early stage of a project and decreases with time. Furthermore, our results show that there are both synergistic effect and substitution effect among different signals. Specifically, the direction of interaction effect depends on the forms of signals and the backers’ sensitivity toward that signal, and the interaction effects are also dynamic. |
| 11 | 2020 | Journal of Business Research | How signal intensity of behavioral orientations affects crowdfunding performance: The role of entrepreneurial orientation in crowdfunding business ventures | We show that signals of autonomy, innovativeness, competitive aggressiveness, and risk-taking have an inverted-U-shaped relationship with crowdfunding performance. Signals of proactiveness have a positive non-monotonic relationship with crowdfunding performance. |
| 12 | 2020 | ICIS 2020 | It's all in the (Sub-)title? Expanding Signal Evaluation in Crowdfunding Research | We find that incorporating subtitle information increases the variance explained by the respective models and therefore their predictive capability for funding success. |
| 13 | 2020 | Journal of Risk and Financial Management | The effect and impact of signals on investing decisions in reward-based crowdfunding: A comparative study of China and the United Kingdom | The findings reveal that signals actively mitigate the problem of information asymmetry in both countries, but this varies in the sense that higher goal setting has a more positive/impactful relationship with project success in the UK than it does in China. Project comments are more positively associated with project success in China as compared to the UK, whereas project updates are more negatively related to project success in China as compared to the UK. |
| 14 | 2020 | Electronic Commerce Research and Applications | The impact of soft information extracted from descriptive text on crowdfunding performance | the empirical results show that the impacts of ex- perience of a fundraiser, comment quantity, and the number of backers on crowdfunding performance are sig- nificantly positive, whereas reward execution moderates the impact of goal. In addition to the hard information, we find that the soft information also contributes greatly to crowdfunding performance. Specifically, the topics of project descriptions play an important role in crowdfunding performance. The positive sentiment in project comments has a positive impact on crowdfunding performance, whereas the negative one has a negative impact |
| 15 | 2019 | Electronic Markets | The recipe of successful crowdfunding campaigns | Our findings reveal that not only the signals themselves but also their interrelations are important to explain funding success. |
| 16 | 2018 | Procedia computer science | Empirical Analysis of Signals on Crowdfunding with Trust Theory | In the attractive process of quality signals, the number of project pictures and the minimum investment amount are significant and positively affecting the successful financing of the project. Whether the project description has video and number of return levels is not significant, but they have a positive impact on successful financing. In the interactive trust process, the number of updates and comments in the project are very significant, which indicate that the investor trust is a key factor in promoting the investor funds to support the project. In the process of investor funds to support project, both the number of backers and the supporters of minimum investment significantly affect successful financing, and the amount of financing goal significantly negatively influences the financing results. |
| 17 | 2018 | Journal of Business Research | Exploring the multi-sided nature of crowdfunding campaign success | This study identifies the most appealing signals and analyzes the results through a multi-theory approach as follows. First, contrary to altruism and warm-glow giving theories, crowdfunding to support social impact projects does not perform well. Second, fixed campaigns, small-sized projects, and prolonged campaigns are more likely to be funded, in line with goal-setting theory. Additionally, the resource-based view of firms clarifies the importance of having large entrepreneurial teams. Further, narrative theories about persuasion and information processing provide insights into why detailed text descriptions are more persuasive than pitch videos. Finally, this study relies on social identity and signaling theories to illustrate the impressive role of social networks and discussion forums between fundraisers and backers for developing effective word-of-mouth. |
| 18 | 2018 | Organization Science | Extending signaling theory to rhetorical signals: Evidence from crowdfunding | We find that rhetorical signals complement substantive signals in certain situations and, thus, strengthen their impact on a firm’s financial resource acquisition. Contrary to our expectations, however, we find that under specific conditions, rhetorical signals may also weaken the impact of substantive signals. |
| 19 | 2018 | Journal of Business Venturing | Signals’ flexibility and interaction with visual cues: Insights from crowdfunding | We find that media coverage signals maintain effectiveness in the crowdfunding context, yet conversely, references to patent ownership are negative or unrelated to crowdfunding performance. These results provide evidence that some signals remain effective (i.e., flexible) across exchange contexts, while others are less flexible. In terms of the interaction of different aspects of entrepreneurs' pitches, we examine entrepreneurs' use of visual cues alongside signals. The presentation quality of the entrepreneurs' pitches. represents a salient visual cue in the crowdfunding context. We find that video quality has a positive effect on crowdfunding performance by shaping impressions held by potential funders. Our results suggest a more nuanced set of interactions between signals and visual cues. Video quality and each of the costly signals positively interact to influence crowdfunding performance. Contrary to our hypotheses, we also find that text quality and costly signals have negative interaction effects with crowdfunding performance. |
| 20 | 2017 | Electronic Commerce Research | An empirical investigation of signaling in reward-based crowdfunding | Our results indicate that social ties, investment preparation and presentation, the supply of multiple rewards as well as endeavors to communicate and interact with the crowd positively influence the probability of success of a reward-based crowd- funding campaign. In contrast, the funding goal, a campaign’s runtime and the estimated time of delivery for the rewards have a negative impact on the successful completion of a campaign. |
| 21 | 2017 | Entrepreneurship Theory and Practice | Resolving information asymmetry: Signaling, endorsement, and crowdfunding performance | We propose that signals through start-up actions (use of media) and characteristics (crowd- funding experience) can mitigate information asymmetry concerns about project quality and founder credibility, enhancing the project’s likelihood of attaining funding. Further, we posit that while start-up–originated signals offset each other’s effects, third-party endorsements (sentiment expressed in backer comments) validate and complement start-up– originated signals. |
| 22 | 2016 | Local Economy and Regional Development | Social identity and signalling success signals in online crowdfunding | This study suggests that funders and backers who identify themselves with the projects in their own social networks are associated with greater pledge/backer ratio. We also find that projects where the fundraiser and its backers exchange more signals in a joint forum, but not signals delivered unilaterally by the fundraiser, have a greater pledge/backer ratio. |
| 23 | 2016 | Decision Support Systems | The emergence and effects of fake social information: Evidence from crowdfunding | Our results show that fake Facebook Likes have a very short-term positive effect on the number of backers funding the respective crowdfunding campaign. However, this short-term peak is followed by an immediate, sharp drop in the number of backers funding the campaign reaching levels that are lower than prior to the occurrence of the non-genuine social information, leading to a total negative effect over time. |
| 24 | 2015 | AIS | How to Crowdfund More A Signaling Perspective | The results indicate that fundraisers’ previous fundraiser experience, backers’ comments, and fundraisers’ replies positively affect fundraising success. |
| 25 | 2018 | Journal of Research in Marketing and Entrepreneurship | Understanding backers’ interactions with crowdfunding campaigns | It is demonstrated that the conceptual framework presented in the manuscript usefully organizes the real-world tactical marketing decisions of a crowdfunding backer while also being readily amendable to integrating theoretical accounts of human behavior from a diverse body of social science literature. Empirically testable propositions are derived from this social science literature and recast into a manner that could be investigated in the crowdfunding context to expand the body of knowledge on this topic. |
