# Supplementary material for: Fundraiser engagement, third-party endorsement and crowdfunding performance: A configurational theory approach
Source: PLoS One. 2024 Aug 15;19(8):e0308717. doi: 10.1371/journal.pone.0308717 (PMC11326654; doi:10.1371/journal.pone.0308717)
Supplement: S2 Table — (DOCX) [file pone.0308717.s002.docx]

**S2 Table. Truth Table for High Crowdfunding Performance (Funding Ratio)**

| K-means | Update | Fundraiser comment | Facebook sharing | Positive comment | Number | Ratio | raw consist | PRI consist | SYM consist |
| --- | --- | --- | --- | --- | --- | --- | --- | --- | --- |
| 0 | 0 | 0 | 1 | 0 | 3 | 0 | 0.743551 | 0.247063 | 0.247063 |
| 1 | 0 | 0 | 1 | 1 | 1 | 0 | 0.879428 | 0.257788 | 0.257787 |
| 0 | 0 | 0 | 0 | 0 | 4 | 0 | 0.732812 | 0.269774 | 0.317884 |
| 1 | 0 | 0 | 0 | 0 | 2 | 0 | 0.822783 | 0.301305 | 0.395548 |
| 0 | 0 | 0 | 0 | 1 | 5 | 0 | 0.770822 | 0.311853 | 0.311853 |
| 1 | 1 | 0 | 0 | 0 | 1 | 0 | 0.845101 | 0.353571 | 0.427877 |
| 0 | 0 | 1 | 1 | 1 | 3 | 0 | 0.788883 | 0.409415 | 0.409415 |
| 1 | 1 | 0 | 1 | 0 | 2 | 0 | 0.867271 | 0.412926 | 0.412926 |
| 0 | 0 | 1 | 0 | 0 | 1 | 0 | 0.85017 | 0.442925 | 0.447397 |
| 0 | 0 | 1 | 1 | 0 | 1 | 0 | 0.867679 | 0.455357 | 0.455357 |
| 0 | 0 | 0 | 1 | 1 | 1 | 0 | 0.776431 | 0.463023 | 0.463023 |
| 0 | 0 | 1 | 0 | 1 | 2 | 0 | 0.834309 | 0.475283 | 0.477496 |
| 1 | 1 | 0 | 0 | 1 | 2 | 0 | 0.872017 | 0.495268 | 0.511123 |
| 1 | 1 | 0 | 1 | 1 | 3 | 0 | 0.881766 | 0.511765 | 0.511765 |
| 1 | 0 | 1 | 0 | 0 | 2 | 0 | 0.901159 | 0.513723 | 0.556826 |
| 0 | 1 | 1 | 1 | 1 | 4 | 1 | 0.932706 | 0.751149 | 0.803371 |
| 0 | 1 | 1 | 0 | 0 | 3 | 1 | 0.935191 | 0.756691 | 0.761322 |
| 0 | 1 | 1 | 0 | 1 | 3 | 1 | 0.927592 | 0.763933 | 0.788389 |
| 0 | 1 | 0 | 1 | 1 | 1 | 1 | 0.951288 | 0.813085 | 0.813084 |
| 1 | 1 | 1 | 1 | 1 | 8 | 1 | 0.957334 | 0.824675 | 0.880277 |
| 0 | 1 | 0 | 1 | 0 | 1 | 1 | 0.971862 | 0.845727 | 0.863946 |
| 1 | 1 | 1 | 0 | 1 | 2 | 1 | 0.984869 | 0.943299 | 0.943299 |
| 1 | 1 | 1 | 1 | 0 | 1 | 1 | 0.996353 | 0.953771 | 0.975124 |
| 1 | 1 | 1 | 0 | 0 | 3 | 1 | 0.99713 | 0.978235 | 0.988426 |
