# Supplementary material for: Fundraiser engagement, third-party endorsement and crowdfunding performance: A configurational theory approach
Source: PLoS One. 2024 Aug 15;19(8):e0308717. doi: 10.1371/journal.pone.0308717 (PMC11326654; doi:10.1371/journal.pone.0308717)
Supplement: S3 Table — (DOCX) [file pone.0308717.s003.docx]

**S3 Table. Truth Table for High Crowdfunding Performance (Success)**

| K-means | Update | Fundraiser  comment | Facebook  sharing | Positive  comment | Number | Ratio | raw consist | PRI consist | SYM consist |
| --- | --- | --- | --- | --- | --- | --- | --- | --- | --- |
| 1 | 1 | 1 | 0 | 1 | 2 | 1 | 0.883081 | 0.883081 | 0.883081 |
| 1 | 1 | 1 | 1 | 1 | 8 | 1 | 0.872145 | 0.872145 | 0.872145 |
| 0 | 1 | 1 | 1 | 1 | 4 | 1 | 0.866832 | 0.866832 | 0.866832 |
| 0 | 1 | 1 | 0 | 1 | 3 | 1 | 0.864538 | 0.864538 | 0.864538 |
| 0 | 1 | 1 | 0 | 0 | 3 | 1 | 0.863902 | 0.863902 | 0.863902 |
| 0 | 1 | 0 | 1 | 0 | 1 | 1 | 0.856275 | 0.856275 | 0.856275 |
| 0 | 1 | 0 | 1 | 1 | 1 | 1 | 0.853862 | 0.853862 | 0.853862 |
| 1 | 1 | 1 | 0 | 0 | 3 | 1 | 0.852654 | 0.852654 | 0.852654 |
| 1 | 1 | 1 | 1 | 0 | 1 | 1 | 0.851369 | 0.851369 | 0.851369 |
| 0 | 0 | 1 | 0 | 0 | 1 | 0 | 0.776457 | 0.776457 | 0.776457 |
| 0 | 0 | 1 | 1 | 0 | 1 | 0 | 0.750542 | 0.750542 | 0.750542 |
| 1 | 1 | 0 | 0 | 0 | 1 | 0 | 0.749786 | 0.749786 | 0.749786 |
| 0 | 0 | 1 | 0 | 1 | 2 | 0 | 0.747967 | 0.747967 | 0.747967 |
| 1 | 1 | 0 | 0 | 1 | 2 | 0 | 0.742568 | 0.742568 | 0.742568 |
| 1 | 1 | 0 | 1 | 1 | 3 | 0 | 0.740741 | 0.740741 | 0.740741 |
| 1 | 0 | 1 | 0 | 0 | 2 | 0 | 0.738092 | 0.738092 | 0.738092 |
| 1 | 0 | 0 | 0 | 0 | 2 | 0 | 0.736546 | 0.736546 | 0.736546 |
| 1 | 1 | 0 | 1 | 0 | 2 | 0 | 0.7225 | 0.7225 | 0.7225 |
| 1 | 0 | 0 | 1 | 1 | 1 | 0 | 0.694469 | 0.694469 | 0.694469 |
| 0 | 0 | 1 | 1 | 1 | 3 | 0 | 0.693694 | 0.693694 | 0.693694 |
| 0 | 0 | 0 | 0 | 0 | 4 | 0 | 0.672429 | 0.672429 | 0.672429 |
| 0 | 0 | 0 | 0 | 1 | 5 | 0 | 0.670744 | 0.670744 | 0.670744 |
| 0 | 0 | 0 | 1 | 1 | 1 | 0 | 0.666378 | 0.666378 | 0.666378 |
| 0 | 0 | 0 | 1 | 0 | 3 | 0 | 0.605601 | 0.605601 | 0.605601 |
